# Tripeptidyl Peptidase II Regulates Sperm Function by Modulating Intracellular Ca^2+^ Stores via the Ryanodine Receptor

**DOI:** 10.1371/journal.pone.0066634

**Published:** 2013-06-20

**Authors:** Yuchuan Zhou, Yanfei Ru, Chunmei Wang, Shoulin Wang, Zuomin Zhou, Yonglian Zhang

**Affiliations:** 1 Shanghai Key Laboratory for Molecular Andrology, State Key Laboratory of Molecular Biology, Institute of Biochemistry and Cell Biology, Shanghai Institutes for Biological Sciences, Chinese Academy of Sciences, Shanghai, China; 2 State Key Laboratory of Reproductive Medicine, Institute of Toxicology, School of Public Health, Nanjing Medical University, Nanjing, Jiangsu, China; 3 State Key Laboratory of Reproductive Medicine, Department of Histology and Embryology, Nanjing Medical University, Nanjing, Jiangsu, China; 4 Shanghai Institute of Planned Parenthood Research, Shanghai, China; Institute of Zoology, Chinese Academy of Sciences, China

## Abstract

Recent studies have identified Ca^2+^ stores in sperm cells; however, it is not clear whether these Ca^2+^ stores are functional and how they are mobilized. Here, in vitro and in vivo, we determined that tripeptidyl peptidase II antagonists strongly activated the cAMP/PKA signaling pathway that drives sperm capacitation-associated protein tyrosine phosphorylation. We demonstrated that in the absence of Ca^2+^, TPIII antagonists elevated the intracellular Ca^2+^ levels in sperm, resulting in a marked improvement in sperm movement, capacitation, acrosome reaction, and the in vitro fertilizing ability. This antagonist-induced release of intracellular Ca^2+^ could be blocked by the inhibitors of ryanodine receptors (RyRs) which are the main intracellular Ca^2+^ channels responsible for releasing stored Ca^2+^. Consistent with these results, indirect immunofluorescence assay using anti-RyR antibodies further validated the presence of RyR_3_ in the acrosomal region of mature sperm. Thus, TPPII can regulate sperm maturation by modulating intracellular Ca^2+^ stores via the type 3 RyR.

## Introduction

Mammalian sperm must undergo functional alterations after maturation in the epididymis before they can competently interact with oocytes. This process is referred to as capacitation. Cauda epididymal and ejaculated sperm can be capacitated both in the female reproductive tract and in chemically defined media. Nevertheless, caput epididymal sperm do not possess the ability to undergo capacitation and fertilize eggs [1], [2].

Sperm capacitation comprises a series of processes, including modifications in the distribution of surface protein; alterations in the plasma membrane characteristics; changes in enzymatic activities; modulation of intracellular constituents such as cyclic adenosine monophosphate (cAMP), Ca^2+^, and HCO_3_^–^; and protein tyrosine phosphorylation [3]. With respect to these changes, it is important to mention that protein tyrosine phosphorylation is closely correlated to sperm capacitation [2], [4]. Furthermore, in many mammalian species, protein tyrosine phosphorylation is considered an indicator of sperm capacitation and is associated with hyperactivated motility, zona pellucida binding, and acrosome reaction [5]–[7]. It is widely accepted that sperm protein tyrosine phosphorylation is regulated by the soluble adenylyl cyclase (sAC)/cAMP/protein kinase A (PKA) signaling pathway [3]; but the mechanism by which the cascade of this signaling pathway is activated remains unclear.

Ca^2+^ signaling in sperm is critical for fertilization, and it plays a pivotal role in sperm maturation, including motility, capacitation, and acrosome reaction [1], [8]. Impaired Ca^2+^ signaling in sperm is associated with male subfertility [9], [10]. Ca^2+^ can directly stimulate adenylyl cyclase, leading to the activation of the sAC/cAMP/PKA signaling pathway in sperm [5], [11]. However, the function of the Ca^2+^ stores in mature sperm is not well understood, and the mechanism by which intracellular Ca^2+^ stores are mobilized remains to be elucidated.

Several studies have indicated that some proteolytic enzymes are closely associated with sperm maturation and calcium signaling. Calpain, a cysteine protease, has been shown to modulate sperm capacitation and acrosome reaction in association with extracellular Ca^2+^ [12]. Metalloendoprotease inhibitors could block acrosome reaction and the increased intracellular Ca^2+^ levels in human spermatozoa induced by follicular fluid [13]. Trypsin inhibitors prevent the progesterone-initiated increase in human sperm intracellular calcium [14]. A previous study on sea urchins revealed that activation of the Ca^2+^ channels during the acrosome reaction in sperm was found to be repressed by inhibitors of chymotrypsin-like proteases [15]. In addition, increasing evidence demonstrates that sperm proteasomes play an active role during the zona pellucida- and progesterone-induced acrosome reaction and the calcium influx [16], [17].

Recently, it was found that tripeptidyl peptidase II (TPPII) could operate mostly downstream of proteasomes in cytosolic proteolysis [18]–[20]. TPPII is able to protect cells under conditions of cellular stress. For example, it is up-regulated in lymphoma cells adapted to grow in the presence of proteasome inhibitors [18], [20], [21]. TPPII also plays a critical role in several vital cellular processes such as antigen processing, apoptosis, DNA damage repair, or cell division, and is also involved in muscle wasting, obesity, and cancer [22], [23]. In vivo, various phenotypes of different TPPII-deficient mice have been reported. Mice that were homozygous for an insertion in the *Tpp2* locus could not be obtained due to early embryonic lethality. However, Their *Tpp2* heterozygotes were leaner than their wild-type littermates, while their food intake was normal [24]. Gene-trapped disrupting *Tpp2* mice with >90% reduced expression of TPPII compared to the wild-type mice were viable, fertile, and normal in appearance and behavior [25]. In contrast, knockout mice homozygotic for *Tpp2* ^−/−^ were viable and grossly indistinguishable from wide type littermates, but in these mice, TPPII deficiency activated cell type-specific death programs [26]. It is unclear why there is such major difference in the different TPPII- deficient mice. TPPII is an evolutionarily conserved serine peptidase of the subtilisin family [18], [27]. Previous reports have indicated that TPPII is widely found in eukaryotic cells in a variety of tissues. It is most highly expressed in the testis [28], [29]. However, it is still unknown whether TPPII plays any role in the reproductive system. Thus far, the knowledge of molecular mechanisms for the action of TPPII is largely limited.

Our present work by using a pharmacological approach aimed to investigate the role of TPPII in sperm maturation and to identify the mechanisms by which it regulates sperm function.

## Results

### Characteristics of TPPII Protein in Sperm

**Figure 1:**
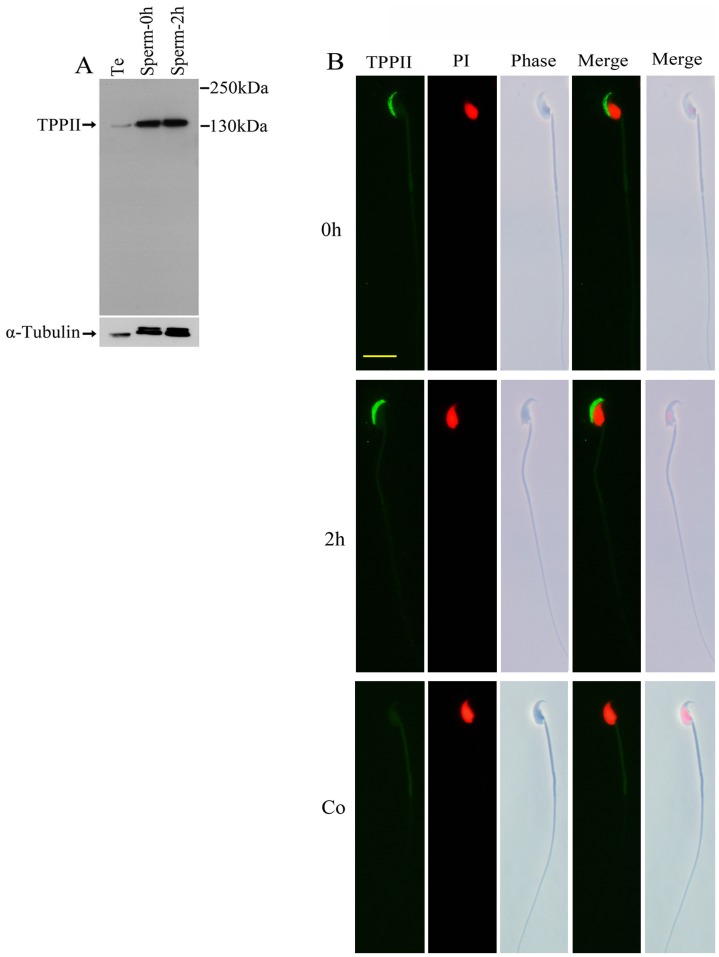
Characteristics of TPPII protein in mouse sperm. (A) Western blot analysis of TPPII protein in testis tissue (Te) and cauda epididymal sperm incubated in EKRB medium for 0 (Sperm-0 h) and 2 (Sperm-2 h) h. The blot was probed with monoclonal antibodies against α-tubulin to assess protein loading. The Western blot is a representative of three independent experiments. (B) Immunofluorescence staining of TPPII protein on spermatozoa. Cauda sperm before (0 h) and after (2 h) capacitation were probed with anti-TPPII polyclonal antibodies. Control sperm (Co) were examined by anti-TPPII polyclonal antibodies which were pre-incubated with the corresponding antigen peptide. Sperm DNA was stained with propidium iodide (PI) and can be seen in red. (bars 10 µm). A representative of three independent experiments is shown.

TPPII was widely discovered in a variety of tissues, including the testes, brain, spleen, lungs, liver, heart, and kidneys [28], [29]. The dominant form of TPPII is extralysosomal and soluble. TPPII in liver was considered as purely cytosolic [30]; whereas its membrane-associated variant was detected in the brain and the testis [28], [31]. The highest level of membrane-bound and soluble TPPII catalytic activity was present in the testis alone [28]. However, thus far, none of the studies have reported the characteristics and localization of TPPII in sperm. Here, western blot analysis confirmed that the positive TPPII signal was expressed in the testis and was also present in the sperm before and after capacitation (Figure 1A). Immunofluorescence assay further validated that the TPPII protein is located on the region of sperm acrosome before and after sperm capacitation (Figure 1B; Also see Figure S1).

### TPPII Antagonists can Accelerate Sperm Capacitation-associated Protein Tyrosine Phosphorylation

**Figure 2:**
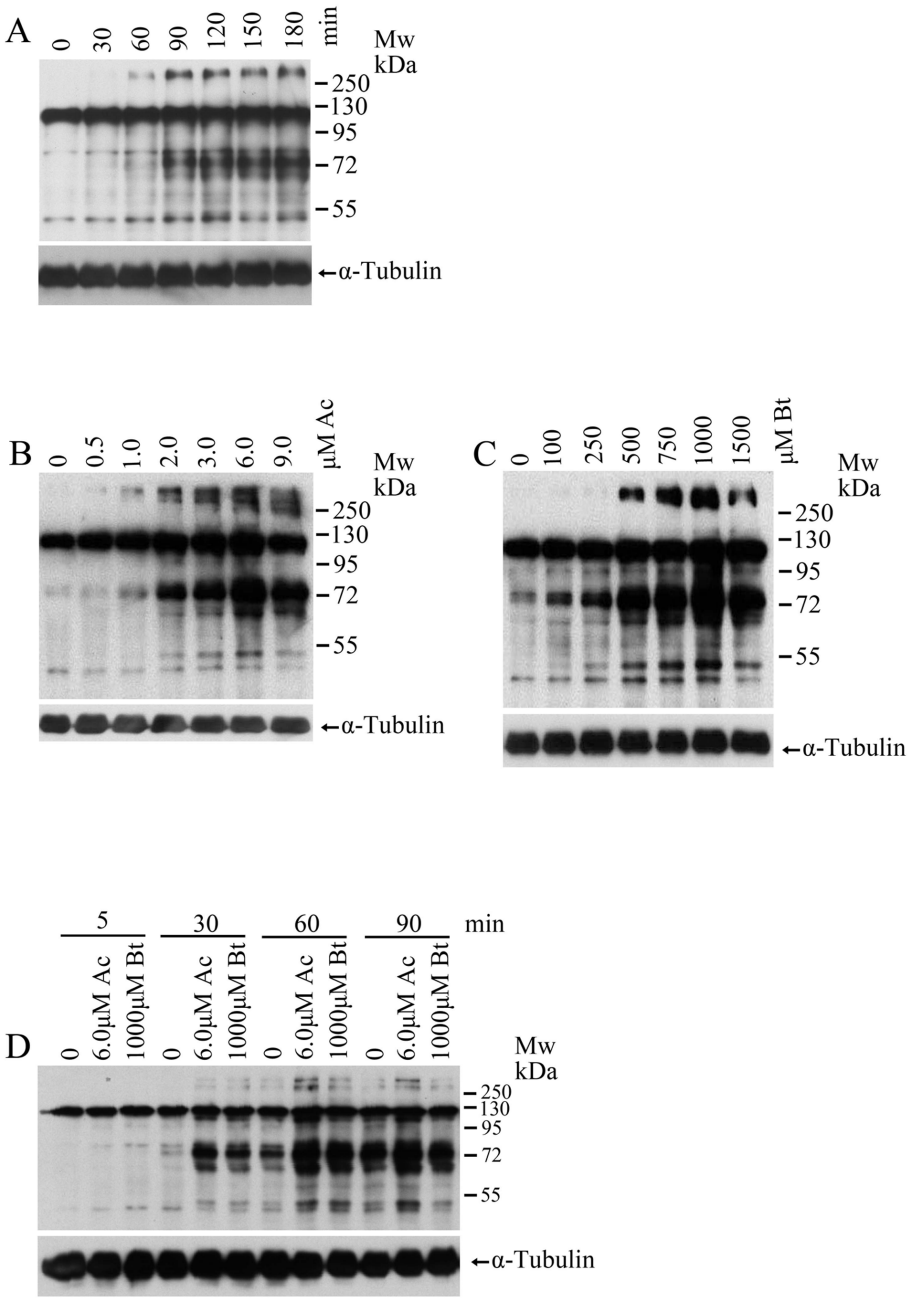
Effects of two different TPPII antagonists on sperm capacitation-associated protein tyrosine phosphorylation. (A) Time-dependent protein tyrosine phosphorylation of mouse spermatozoa. Cauda epididymal spermatozoa were incubated in EKRB medium and collected at 0, 30, 60, 90, 120, 150, and 180 minutes after incubation. α-Tubulin was used as the loading control. The Western blot is a representative of five independent experiments. (B,C) Dose-dependent effects of TPPII antagonists on sperm protein tyrosine phosphorylation. Sperm were incubated with the TPPII antagonists AAF-CMK (B)(Ac: 0, 0.5, 1.0, 2.0, 3.0, 6.0 and 9.0 µM) and butabindide (C)(Bt: 0, 100, 250, 500, 750, 1000 and 1500 µM) for one hour. Protein tyrosine phosphorylation was assessed by Western blot analysis. α-Tubulin was used as the loading control. The Western blot is a representative of five independent experiments. (D) Time-dependent effects of TPPII antagonists on sperm protein tyrosine phosphorylation. Spermatozoa were treated by TPPII antagonists, AAF-CMK (Ac, 6 µM), and butabindide (Bt, 1000 µM) for 5, 30, 60, and 90 minutes. Protein tyrosine phosphorylation was assessed by Western blot analysis. α-Tubulin was used as the loading control. The Western blot is a representative of five independent experiments.

Since TPPII shows enzymatic activity, we considered that it may play an important role in sperm maturation, and investigated its function using two TPPII antagonists–butabindide and AAF-CMK. Butabindide is a reversible and the most specific TPPII antagonist [28], whereas AAF-CMK is an irreversible and chemically more stable than butabindide [18]. To test the effect of TPPII antagonists on sperm maturation in vitro, we used EKRB solution as the capacitation medium. Figure 2A shows a time-dependent increase in sperm capacitation-associated tyrosine phosphorylation. These results are in agreement with the previously published findings in mouse studies [2], [7]. Thus, by using this culture system, we found that TPPII antagonists accelerated protein tyrosine phosphorylation in the dose- and time-dependent manners (Figure 2B, 2C and 2D). The tyrosine phosphorylation reached the highest levels when incubated for 60 min with 6 µM AAF-CMK and 1000 µM butabindide. Unless otherwise noted, we used these two concentrations of the two antagonists and the incubation time of 60 min for the subsequent experiments in this study. Our data demonstrated that TPPII antagonists could significantly accelerate the sperm capacitation-associated tyrosine phosphorylation.

### TPPII Antagonists Activates Sperm sAC/cAMP/PKA Pathway in the Absence of Extracellular Ca^2+^

**Figure 3:**
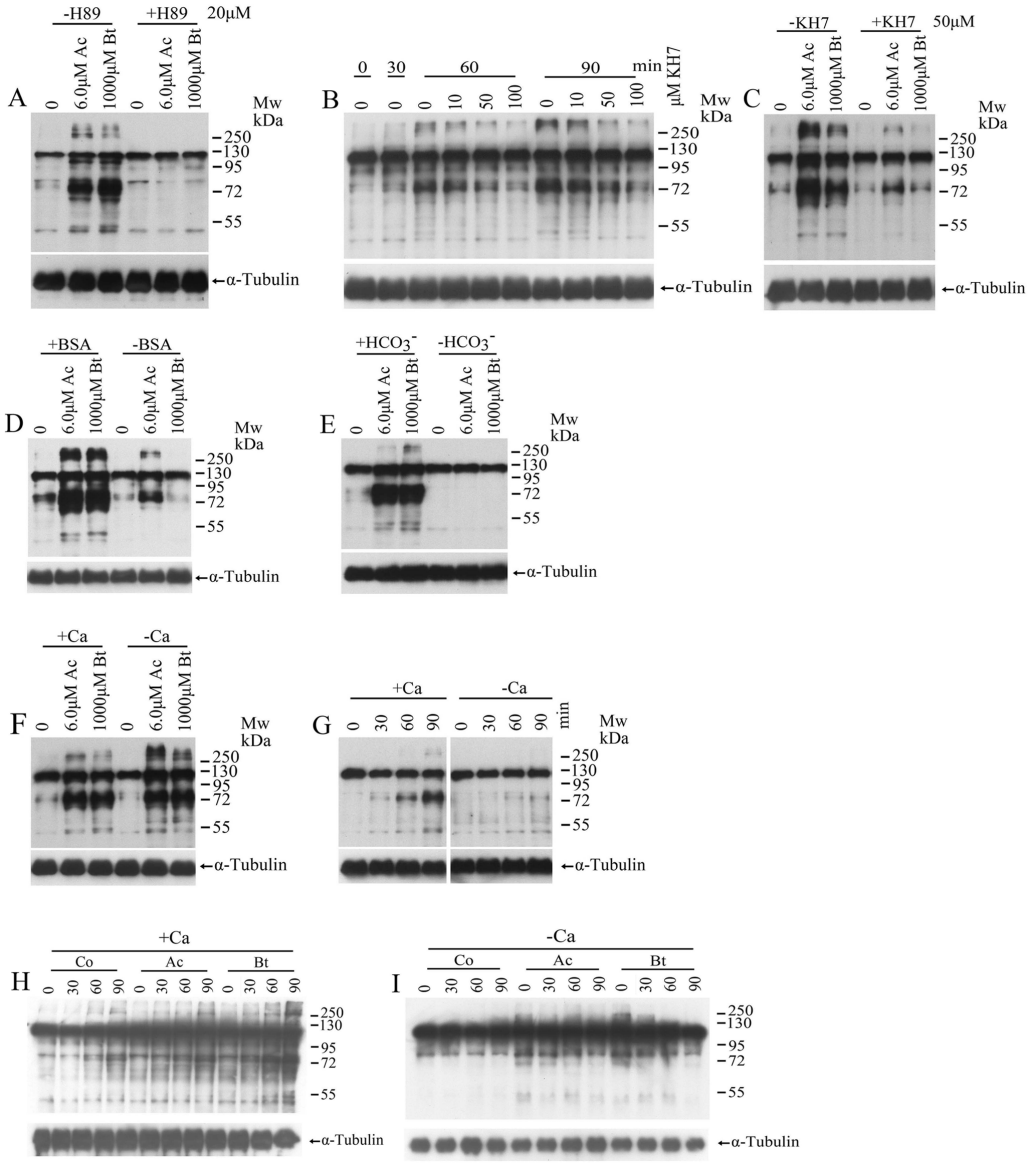
TPPII antagonists activate sperm sAC/cAMP/PKA pathway. (A) Spermatozoa were treated with the TPPII antagonists AAF-CMK (Ac, 6 µM) and butabindide (Bt, 1000 µM) for 60 min in the absence or presence of 20 µM the protein kinase A (PKA) inhibitor H89. Protein tyrosine phosphorylation was then assessed by Western blot analysis. α-Tubulin was used as the loading control. The Western blot is a representative of five independent experiments. (B) Spermatozoa were incubated in the medium with different concentration of soluble adenylyl cyclase (sAC) blocker KH7 (0, 10, 50, and 100 µM) for different durations (0, 30, 60, and 90 minutes). Protein tyrosine phosphorylation was assessed by Western blot analysis. α-Tubulin was used as the loading control. The Western blot is a representative of five independent experiments. (C) Spermatozoa were treated by AAF-CMK (Ac, 6 µM) and butabindide (Bt, 1000 µM) for 60 min in the absence or presence of 50 µM KH7. Protein tyrosine phosphorylation was then assessed by Western blot analysis. α-Tubulin was used as the loading control. The Western blot is a representative of five independent experiments. (D) Sperm were treated with AAF-CMK (Ac, 6 µM) and butabindide (Bt, 1000 µM) for 60 min in the absence or presence of 3 mg/ml BSA. Protein tyrosine phosphorylation was then assessed by Western blot analysis. α-Tubulin was used as the loading control. The Western blot is a representative of five independent experiments. (E) Sperm were treated with AAF-CMK (Ac, 6 µM) and butabindide (Bt, 1000 µM) for 60 min in the absence or presence of 25 mM HCO_3_^–^. Protein tyrosine phosphorylation was then assessed by Western blot analysis. α-Tubulin was used as the loading control. The Western blot is a representative of five independent experiments. (F) Sperm were treated with AAF-CMK (Ac, 6 µM) and butabindide (Bt, 1000 µM) for 60 min in the absence or presence of 1 mM CaCl_2_. Protein tyrosine phosphorylation was assessed by Western blot analysis. α-Tubulin was used as the loading control. The Western blot is a representative of five independent experiments. (G) Time course of capacitation-associated protein tyrosine phosphorylation of spermatozoa in the absence or presence of 1 mM CaCl_2_. Protein tyrosine phosphorylation was assessed by Western blot analysis. α-Tubulin was used as the loading control. The Western blot is a representative of five independent experiments. (H,I) The two TPPII antagonists were injected into the tail vein at 10 mg/kg and 10 µg/kg, respectively, according to body weight. After one hour, cauda sperm from the treated mice were collected and incubated for different times in the presence (h) or absence (i) of Ca^2+^. Protein tyrosine phosphorylation was assessed by Western blot analysis. α-Tubulin was used as the loading control. The Western blot is a representative of five independent experiments.

It is widely accepted that sperm capacitation-associated protein tyrosine phosphorylation is regulated by the sAC/cAMP/PKA pathway. The presence of BSA, Ca^2+^, and HCO_3_^–^ in the medium is essential for the activation of this pathway [2]. To test whether the effects of TPPII antagonists on tyrosine phosphorylation was related to the sAC/cAMP/PKA pathway, we used a highly selective blocker (H89) of PKA and a specific inhibitor (KH7) of sAC. The results showed that H89 and KH7 all suppressed the increase of protein tyrosine phosphorylation stimulated by TPPII antagonists (Figure 3A, 3B and 3C). Furthermore, we examined the effect of TPPII antagonists on tyrosine phosphorylation of sperm incubated in media devoid of BSA, HCO_3_^–^, and Ca^2+^. When the sperm were incubated in the absence of BSA or HCO_3_^–^ for one hour, the acceleration of tyrosine phosphorylation induced by TPPII antagonists disappeared (Figure 3D and 3E). This demonstrates that the effect of TPPII antagonists on tyrosine phosphorylation is dependent on the presence of BSA and HCO_3_^–^ in the medium. As illustrated in Figure 3F, TPPII antagonists could still accelerate tyrosine phosphorylation in the absence of extracellular Ca^2+^, although Ca^2+^ in the medium is also essential for tyrosine phosphorylation (Figure 3G). In vivo, we injected TPPII antagonists into the tail vein, collected the cauda epididymal sperm and examined the change of protein tyrosine phosphorylation of sperm incubate in media with and without Ca^2+^. The results revealed that the protein tyrosine phosphorylation of sperm from mice treated by TPPII antagonists was accelerated regardless of the presence or absence of Ca^2+^ in the medium (Figure 3H and 3I). This suggests that stimulation of TPPII antagonists on tyrosine phosphorylation is independent of extracellular Ca^2+^ in the medium in vitro and in vivo.

### TPPII Antagonists Regulates Sperm Function by Modulating Intracellular Calcium

**Figure 4:**
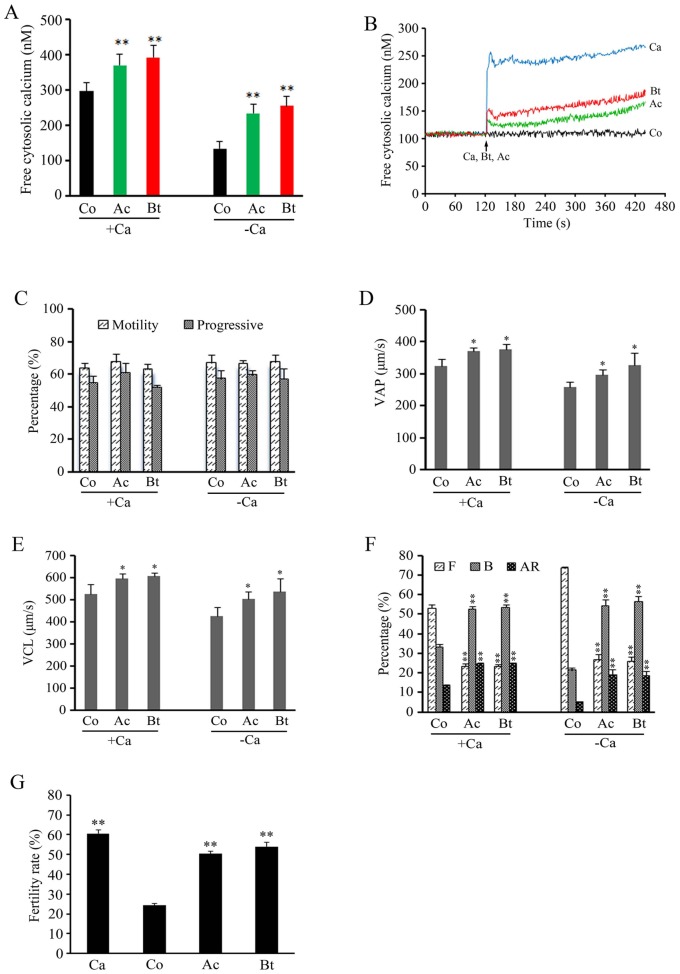
TPPII antagonists regulate sperm function by increasing intracellular calcium. (A) Spermatozoa were treated with AAF-CMK (Ac, 6 µM) and butabindide (Bt, 1000 µM) for 60 min in the absence or presence of 1 mM CaCl_2_ and the intracellular Ca^2+^ level in the sperm was examined. Results are expressed as the mean ± SEM (n = 7). **P<0.001, as compared with the corresponding controls (Co) (unpaired *t* test). (B) Effect of AAF-CMK (Ac, 6 µM) and butabindide (Bt, 1000 µM) on the release of sperm intracellular Ca^2+^. The arrow indicates the time points at which the calcium iron or antagonists were added. A representative of four experiments is presented. Control (Co) is the basal level. The final concentration of calcium (Ca) is 1 mM, obtained by the addition of 100 mM CaCl_2_ solution. (C–E) Spermatozoa were treated with AAF-CMK (Ac, 6 µM) and butabindide (Bt, 1000 µM) for 60 min in the absence or presence of 1 mM CaCl_2_. The percentage (C), VAP (D), and VCL (E) of sperm motility were examined using CASA. Results are expressed as the mean ± SEM (n = 4). *P<0.05 as compared with the corresponding control (Co) (unpaired *t* test). (F) Spermatozoa were treated with AAF-CMK (Ac, 6 µM) and butabindide (Bt, 1000 µM) for 60 min in the absence or presence of 1 mM CaCl_2_. The capacitation and acrosome reaction were assessed by chlortetracycline (CTC) fluorescence. F: uncapacitated sperm with intact acrosome; B: capacitated sperm with intact acrosome; AR: capacitated sperm with reacted acrosome. Results are expressed as the mean ± SEM (n = 6). **P<0.001, compared with the control (Co) (unpaired *t* test). (G) Spermatozoa were treated with AAF-CMK (Ac, 6 µM) and butabindide (Bt, 1000 µM) for 60 min in complete medium without 1 mM CaCl_2_. Sperm-egg fertilization was performed as described in Methods. Data are reported as mean ± SEM of three independent experiments. **P<0.001, as compared with the corresponding control (Co) (unpaired *t* test).

The presence of extracellular free Ca^2+^ in the medium is essential for sperm protein tyrosine phosphorylation [2]. However, our results indicated that TPPII antagonists significantly accelerated protein tyrosine phosphorylation when sperm were incubated in a Ca^2+^-deficient medium. We hypothesized that intracellular Ca^2+^ in sperm is elevated by TPPII antagonists. To test this hypothesis, we investigated the effect of TPPII antagonists on intracellular Ca^2+^ in sperm. The two antagonists were directly added to the sperm suspension and incubated for one hour. The result showed that both antagonists significantly increased the sperm intracellular Ca^2+^ level regardless of the presence of Ca^2+^ in the medium (Figure 4A). Moreover, as shown in Figure 4B, the antagonist-induced increase in Ca^2+^ level was fast. The free cytosolic calcium in the sperm was continually elevated since the TPPII antagonists were added into the Ca^2+^-free medium. The TPPII antagonist-induced pattern of intracellular calcium change was the same as that of extracellular Ca^2+^ stimulation. These indicated that TPPII antagonists can promote sperm protein tyrosine phosphorylation by mobilizing the intracellular Ca^2+^ ions in sperm.

Our further results illustrated that TPPII antagonist-induced increase of sperm intracellular Ca^2+^ did not have any obvious effects on the percentage of motility and progressive motility (Figure 4C), but it resulted in significantly elevated levels of curvilinear velocity (VCL) and average path velocity (VAP) (Figure 4D and 4E). CTC analysis showed that TPPII antagonists obviously increased the percentage of B and AR patterns in sperm incubated in the absence and presence of extracellular Ca^2+^ (Figure 4F), confirming that TPPII antagonists significantly facilitated sperm motility, capacitation, and acrosome reaction by modulating the intracellular Ca^2+^.

The extracellular free calcium ions are vital for successful sperm-egg fertilization in mammals. Mouse sperm that were pre-incubated in a Ca^2+^-deficient medium have been found to be functionally incompetent, although they gradually acquire fertilizing ability if Ca^2+^ is added at the end of the pre-incubation period [32]. Here, we tested whether the increased intracellular Ca^2+^ levels induced by TPPII antagonists could ameliorate the fertilizing ability of sperm incubated in Ca^2+^-deficient medium. TPPII antagonists were able to significantly increase the rate of fertilization (Figure 4G), indicating that TPPII antagonists could compensate the in vitro fertilizing ability of sperm in the absence of extracellular Ca^2+^ by mobilization of intracellular Ca^2+^.

### Acceleration of TPPII Antagonist-induced Sperm Protein Tyrosine Phosphorylation can be Blocked by RyR Inhibitors

**Figure 5:**
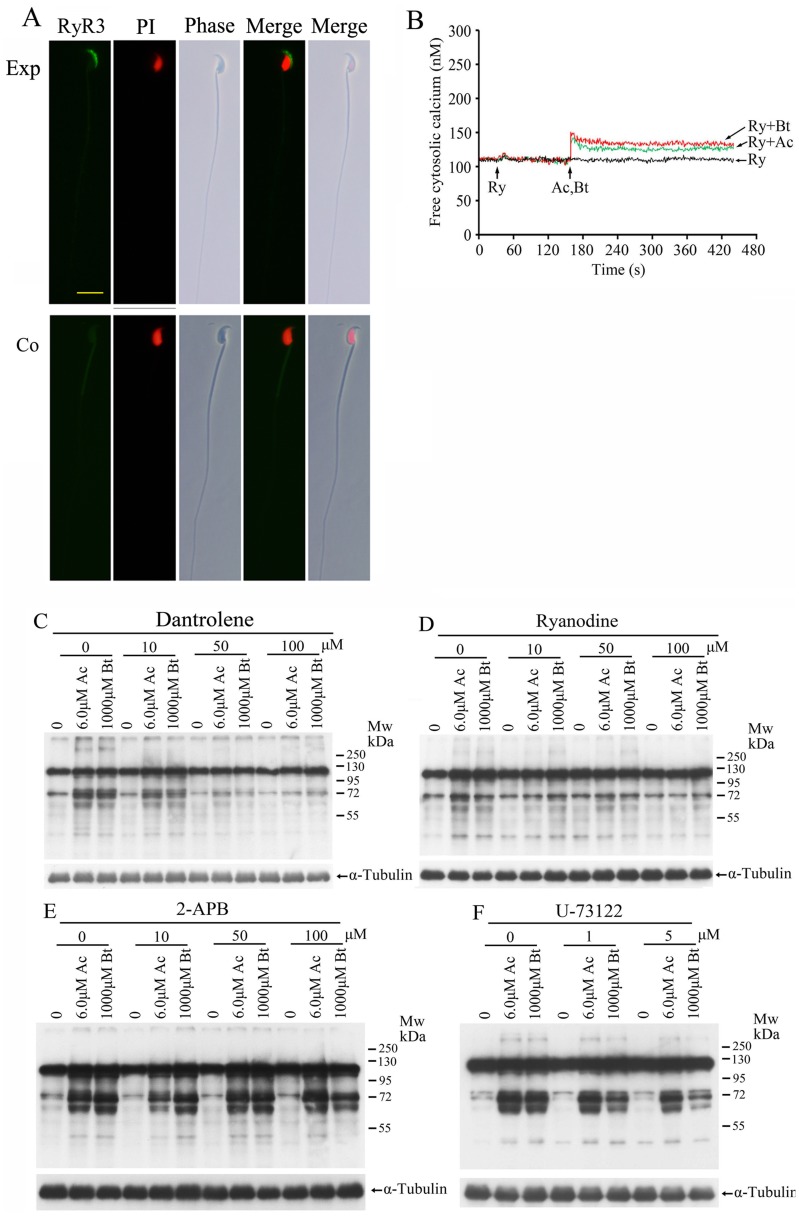
TPPII regulates sperm function by modulating intracellular Ca^2+^ stores via the ryanodine receptor 3. (A) Localization of RyR_3_ protein in spermatozoa. Spermatozoa were probed with (RyR_3_) anti-RyR_3_ and (Co) anti-RyR_3_ pre-incubated with the corresponding antigen peptide. Sperm DNA was stained with propidium iodide (PI) and can be seen in red (bars 10 µm). A representative experiment of three experiments is shown. (B) Release of the intracellular Ca^2+^ stores in sperm was induced by AAF-CMK (Ac, 6 µM) and butabindide (Bt, 1000 µM) in the presence of ryanodine (Ry, 100 µM). Up arrows indicate the time points at which the inhibitors or antagonists were added. A representative experiment of five experiments is shown. (C,D) Sperm were treated with AAF-CMK (Ac, 6 µM) and butabindide (Bt, 1000 µM) for 60 min in the presence of RyR inhibitor dantrolene (C) and ryanodine (D). Protein tyrosine phosphorylation was assessed by Western blot analysis. α-Tubulin was used as the loading control. The Western blot is a representative of five independent experiments. (E,F) Sperm were treated for 60 min with TPPII antagonists AAF-CMK (Ac, 6 µM) and butabindide (Bt, 1000 µM) in the presence of IP_3_R pathway inhibitor 2-APB (E) and U-73122 (F). Protein tyrosine phosphorylation assessed by Western blot analysis. α-Tubulin was used as the loading control. The Western blot is a representative of five independent experiments.

RyRs are intracellular Ca^2+^-release channels. Spermatogenic cells express transcripts for all three RyR isoforms. However, there is no consensus regarding the presence and exact localization of RyRs in mature sperm. Several studies have documented the existence of RyRs in the sperm [33], [34]; contrarily, some studies have reported the absence of RyRs in sperm [35], [36]. To characterize the localization of RyRs on the sperm, indirect immunofluorescence labeling was performed using polyclonal anti-RyR_S_ antibodies. Immunostaining revealed the presence of RyR_3_ on the acrosomal region (Figure 5A; Also see Figure S2). Based on the localization of RyR_3_ and TPPII on the acrosome of the mature sperm, we investigated the role of RyR_3_ in the mechanism of TPPII antagonists. As shown in Figure 5B, the ryanodine of RyR inhibitor could block the TPPII antagonist-induced increase in the intracellular Ca^2+^ concentration. Further investigation indicated that the increase of tyrosine phosphorylation induced by TPPII antagonists was inhibited by prior addition of two RyR inhibitors (Figure 5C and 5D). Inositol 1,4,5-triphosphate receptors (IP_3_Rs) function as another major intracellular Ca^2+^ channel. In mammalian sperm, IP_3_Rs have been detected at the acrosome of several species, including mice, and in some cases also in the RNE [37], [38]. However, two IP_3_R pathway inhibitors could not block the increase of tyrosine phosphorylation induced by TPPII antagonists (Figure 5E and 5F). These data suggest that RyR_3_ but not IP_3_R is involved in increased intracellular Ca^2+^ level and the subsequent acceleration of tyrosine phosphorylation induced by TPPII antagonists.

## Discussion

The sperm maturation process is regulated by multiple molecules, including proteins from the testis and epididymis. It is associated with intracellular changes in calcium, bicarbonate, cAMP, and protein tyrosine phosphorylation levels. These changes render sperm fertilization competence. Currently, there is overwhelming evidence indicating that sperm protein tyrosine phosphorylation is associated with hyperactivated motility, zona pellucida binding, and acrosome reaction [5]–[7], [39]. Here, consistent with the increased TPPII antagonist-induced sperm protein tyrosine phosphorylation, sperm capacitation, and acrosome reaction, the sperm motility and IVF significantly increased following treatment with TPII antagonists. Therefore, TPPII can regulate sperm function by changing the level of protein tyrosine phosphorylation. The present study adopted a pharmacological approach to characterize and identify a novel function of TPPII in sperm maturation.

Activation of sperm capacitation-associated tyrosine phosphorylation is involved in the sAC/cAMP/PKA signaling pathway. Ca^2+^, HCO_3_^–^, and BSA in the surrounding medium have been shown to be essential for this process. BSA in the medium is used as a sink to promote the removal of cholesterol from the plasma membrane, and subsequently increase the sperm membrane fluidity. The entry of bicarbonate and calcium from the medium into the sperm cell activates sAC, resulting in elevated cAMP levels, subsequent PKA activation, and protein tyrosine phosphorylation (Figure S3). Our study indicated that extracellular Ca^2+^ is essential for the activation of the sAC/cAMP/PKA pathway (Figure 3G). However, TPPII antagonists accelerated the capacitation-associated tyrosine phosphorylation of sperm incubated in the absence of Ca^2+^. Moreover, this regulation was found to be dependent on sAC and PKA, as well as extracellular BAS and HCO_3_^–^ in the medium. Further investigation demonstrated that TPPII antagonists could accelerate the tyrosine phosphorylation by strongly elevating the intracellular Ca^2+^ levels in sperm incubated in Ca^2+^-free medium. Although containing trace of amounts of Ca^2+^ contributed by the other salts, this medium supports neither complete capacitation nor obvious tyrosine phosphorylation [32] (Figure 3G). To chelate the traces of Ca^2+^ in Ca^2+^-free medium, EGTA at a final concentration of 25 and 50 µM was used. But this treatment could not abolish TPPII antagonist-induced change of sperm function (Figure S4). Whereas, BAPAT-AM, an intracellular Ca^2+^ chelator, could block the TPPII antagonist-induced increase of sperm motility (VAP and VCL) and capacitation-associated protein tyrosine phosphorylation (Figure S5). Therefore, it is reasonable to conclude that TPPII antagonists trigger the intracellular Ca^2+^ signal to stimulate the sAC/cAMP/PKA-mediated phosphotyrosine pathway and thereafter accelerate sperm motility and capacitation-associated tyrosine phosphorylation. We found that RyR_3_ was localized on the acrosome region of sperm, whereas, RyR_1_ and RyR_2_ was not detected on the sperm (data not shown). The inhibitors of RyRs could block the elevation of TPPII antagonist-induced protein tyrosine phosphorylation and the increase of intracellular Ca^2+^. Thus, TPPII can modulate the intracellular Ca^2+^ store via ryanodine receptor type 3. These data showed that the release of Ca^2+^ from intracellular Ca^2+^ stores alone is sufficient to initiate sperm capacitation and that the initiation and completion of the capacitated state may be regulated by sperm cell itself. Similar to our findings, it was previously reported that thapsigargin-induced release of intracellular Ca^2+^ is sufficient to initiate hyperactivation of bull sperm that were incubated in a Ca^2+^-free medium [36]. This intrinsic control capacity may be effective for sperm to adapt to extreme environments such as in the absence of Ca^2+^.

Inositol 1,4,5-trisphosphate receptors (IP_3_Rs) are another major intracellular Ca^2+^ release channel in sperm, and IP_3_R-gated intercellular Ca^2+^ stores are related to the regulation of sperm hyperactivated motility [36]. Two IP_3_R pathway inhibitors–U-73122 (inhibitor of the hydrolysis of PIP_2_ to IP_3_) and 2-APB (inhibitor of IP_3_R)–were unable to block the TPPII antagonist-induced protein tyrosine phosphorylation (Figure 5E and 5F). Thus, it was indicated that the release of calcium from intracellular stores in the phospholipase signaling pathway was not involved in TPPII action in sperm. So, the RyR-gated intercellular Ca^2+^ store is functional and important for sperm fertilizing capacity. Moreover, this intracellular Ca^2+^ store could be modulated by TPPII. This hypothesis for TPPII action is summarized in Figure S3.

TPPII is a multiple-purpose peptidase. It plays a role in several vital cellular processes such as intracellular protein degradation, antigen processing, apoptosis, or cell division [22], [23]. In vivo, Gene-trapped disrupting *Tpp2* mice with >90% reduced expression of TPPII compared to the wild-type mice were fertile [25]. Young mice with a ubiquitous TPPII deletion were viable and grossly same as WT littermates [26]. Our in vivo results showed that the protein tyrosine phosphorylation of sperm from mice treated by TPPII antagonists was accelerated regardless of the presence of extracellular Ca^2+^ (Figure 3H and 3I), suggesting that protein tyrosine phosphorylation of TPPII-inhibited sperm could still occur independent of extracellular Ca^2+^. It would be interesting to determine whether the difference in the observed phenotypes among TPPII-deficient mice is related to intracellular Ca^2+^ mobilization and compensation.

Protein degradation controls the lifetime of cells, including germ cells. The requirement of proteasomal proteolysis during fertilization has been determined by the application of various proteasome-specific inhibitors and antibodies. Various proteasomal subunits and associated enzymes have been detected in spermatozoa and localized to the acrosome and other structures in sperm [40]. It has been discovered that some physiological substrates can be degraded by the proteasome-ubiquitin system during fertilization [41]. These suggest that the proteins degradation of gamete by a series of enzymatic reaction is important in the reproductive process. TPPII is an important enzyme with proteolytic action. TPPII may even substitute for the proteasome in the degradation of cell proteins and generation of a small fraction of peptides [18], [20]. TPPII can remove tripeptides sequentially from the free N-terminus of larger peptides. It also exhibits endopeptidase activity towards intact proteins or long polypeptide precursors [27], [28]. The colocalization of TPPII and RyR_3_ on the sperm implies the possibility that TPPII functions in association with RyR_3_ in sperm. However, further investigation is required to verify whether TPPII actually degrades the RyR_3_. Whether TPPII directly regulates or indirectly modulates RyR_3_ by a series of products from TPPII remains to be investigated.

In summary, the evidence presented in this study clearly indicates that sperm TPPII is located on the sperm acrosomal region and is involved in the fertilization process. The increased intercellular Ca^2+^ level induced by TPPII antagonists via RyR_3_ represents a mechanism of activation of sperm. Data from these studies provide insights into the intrinsic control events of mammalian sperm that are required for sperm capacitation and fertilization. These findings also shed light on our understanding of the self-protection of sperm.

## Materials and Methods

### Animals

Mature C57 male mice (age: 10–12 weeks) were purchased from the Animal Center of the Chinese Academy of Sciences (Shanghai, China). They were housed in the animal housing at our institute before manipulation. Food and water were freely available throughout the experiments. All protocols were conducted according to the approval of the Institute Animal Care Committee of Shanghai Institute of Biochemistry and Cell Biology (Permit Number: SYXK2007-0017).

### Detection of TPPII Protein on the Sperm

Western blot analysis of the TPPII protein in spermatozoa was conducted according to a previously described protocol [42]. Briefly, total protein extracts obtained from the testis and spermatozoa from the cauda epididymis were resolved by electrophoresis on 8% sodium dodecyl sulfate (SDS)-polyacrylamide gels, transferred into polyvinylidene fluoride (PVDF) membranes, and probed with goat anti-TPPII polyclonal antibodies (Santa Cruz) (dilution: 1∶500). The bound IgG was detected with donkey anti-goat horseradish peroxidase (HRP; dilution: 1∶10000) (Calbiochem) and developed using ECL Plus (Amersham). Protein was assayed by probing the blots with monoclonal antibodies against α-tubulin (Sigma).

### Immunofluorescence Staining

Immunofluorescence was indirectly detected as described previously [42]. Sperm were washed out from the epididymal cauda and fixed in 4% paraformaldehyde for 30 min, and the 1∶100 diluted goat anti-TPPII polyclonal antibodies (Santa Cruz) and rabbit anti-type 3 RyR polyclonal antibodies (Chemicon) were applied. Then, fluorescein isothiocyanate (FITC)-labeled anti-goat IgG and anti-rabbit IgG (Sigma), respectively, were used as the secondary antibodies (dilution : 1∶500). All the images were taken using a BX51 fluorescence microscope (Olympus).

### Culture Media

Enriched Krebs-Ringer bicarbonate (EKRB) medium was used throughout the study for mouse sperm preparation and capacitation, and the preparation was adopted from previously published reports [7]. The final composition of the medium was 120 mM NaCl; 4.8 mM KCl; 1.0 mM CaCl_2_, 1.2 mM MgSO_4_, 1.2 mM KH_2_PO_4_, 5 mM glucose, 21 mM sodium lactate, 0.25 mM sodium pyruvate, 25 mM NaHCO_3_, and 3 mg/ml bovine serum albumin (BSA). All the chemicals were purchased from Sigma and were of the highest purity available. A tenfold concentrated solution of all the ingredients was first prepared without CaCl_2_, BSA, and NaHCO_3_, sterilized by passage through a 0.22-µm filter, and stored at –20°C in single-use aliquots. Working media were prepared by adding CaCl_2_, NaHCO_3_ and BSA and gassing the medium with a mixture of 5% CO2 and 95% air overnight at pH 7.2–7.4. As described in a previous report [2], in some experiments, medium without NaHCO_3_ was derived by adding 25 mM NaCl instead of 25 mM NaHCO_3_. In some experiments, Ca^2+^- and BSA-free media were used and the Ca^2+^ and BSA were added back to above final concentrations if necessary.

### Preparation of Sperm

The cauda epididymis was excised and freed from the fat pad, blood vessels, and connective tissue. The tissue was then transferred to a dish containing 1 ml EKRB medium prewarmed to 37°C, and cut in several places with iridectomy scissors to release the spermatozoa into the medium. After 5 minutes, the sperm suspension was transferred to a 5 ml centrifuge tube. The final concentration of sperm was adjusted to 3–4×10^6^ cells/ml in appropriate medium and assessed using a computer-assisted semen analysis (CASA) machine. After incubation for various time periods and following treatment with different antagonists or inhibitors, the sperm were concentrated by centrifugation at 6,000 g for 2 min at room temperature, washed in phosphate-buffered saline (PBS) three times, resuspended in Laemmli’s sample buffer without mercaptoethanol, and boiled for 5 minutes. After centrifuging at 6,000 g for 2 min, the supernatant was collected, and 2-mercaptoethanol was added to attain a final concentration of 5%. The sperm extract was either used immediately or stored at –70°C until analysis.

### Western Blot for Tyrosine Phosphorylation

SDS-PAGE was carried out in 12% gel. The sperm extracts were electrophoretically transferred to PVDF membranes (GE Healthcare) in all experiments. The blots were blocked with blocking buffer (150 mM NaCl, 5 mM EDTA, 50 mM Tris-HCl, 0.05% (v/v) Triton X-100, 0.25% (m/v) gelatin; pH 7.5) and probed with a monoclonal antibody against phosphotyrosine (clone 4G10, Millipore) and enhanced chemiluminescence detection using an ECL kit (Amersham). To confirm equal protein loading, the blots were stripped and reprobed with anti-α-tubulin monoclonal antibody (Sigma).

### Sperm Motility Analysis

The analysis procedure was a modification of our previously published method [42]. Sperm motility was assayed using an HTM-TOX IVOS sperm motility analyzer (Rat Head Toxicology, version 12.3A, Hamilton-Thorn Research, MA, USA). The instrument settings were as follows: temperature, 37°C; minimum cell size, five pixels; minimum contrast, 50; minimum static contrast, 25; low VAP cutoff, 20.0; low VSL cutoff, 30.0; threshold straightness, 50%; static head size, 0.3–1.95; static head intensity, 0.5–1.3; and magnification, 0.89. Thirty frames were acquired at a frame rate of 60 Hz. The playback feature was used during analysis to check the accuracy of the method.

### Injection of TPPII Antagonists

We injected 10 mg/kg butabindide and 10 µg/kg Ala-Ala-Phe-chloromethylketone (AAF-CMK) into the tail veins of mice according to their body weights. After one hour, cauda sperm from the treated mice were collected and incubated in the absence or presence of Ca^2+^ for 0, 30, 60, and 90 minutes. The total protein was extracted from these sperm and the changes in protein tyrosine phosphorylation were determined according to the method described above.

### Measurement of [Ca^2+^]_i_ Concentration

Measurements were performed as described elsewhere [5], [43]. Spermatozoa were allowed to disperse from the cauda epididymis into the EKRB medium without Ca^2+^ for 5 minutes, after which the sperm suspensions were loaded with the acetoxy-methyl ester of fura-2 (Fura-2/AM; 3 µM final extracellular concentration) and incubated for 30 min at 37°C and 5% CO_2_. The suspensions were then centrifuged in EKRB medium without Ca^2+^ three times at 300 g for 5 min each time to remove extracellular free Fura-2/AM. The spermatozoa were finally resuspended in fresh medium without Ca^2+^, and sperm aliquots were treated by different antagonists or inhibitors under different experimental conditions and the fluorescence was monitored. Spectrofluorometry was performed in a methylacrylate cuvette with magnetic stirring, and warmed to 37°C in a heated cuvette holder. The fluorescence intensity was measured after equilibration for 2 min on a Varian Cary Eclipse (USA) spectrofluorophotometer following excitation at 340 nm and emission at 510 nm. The [Ca^2+^]_i_ was calculated using the equation [Ca^2+^]_i_ = K_d_ (F – F_min_)/(F_max_ – F), where K_d_ = 224 nM. F_max_ and F_min_ were recorded at the end of the incubation period. F_max_ was determined after the addition of 20 µM digitonin, and F_min_ was determined after addition of 10 mM Tris-EGTA to the cuvette.

### Chlortetracycline (CTC) Fluorescence

Sperm aliquots were treated by TPPII antagonists in the medium for 60 min with or without Ca^2+^ at 37°C in an atmosphere containing 5% CO_2_. At the end of the incubation period, they were collected for staining with chlortetracyclin (CTC) and assessing for sperm capacitation and acrosome reaction as described elsewhere [44], [45]. Briefly, 500 µM CTC solution (Sigma) was prepared on the day of use in a buffer of 20 mM Tris-HCl, 130 mM NaCl, and 5 mM cysteine with the final pH adjusted to 7.8. The solution was wrapped in foil at 4°C until just before use. The sperm suspension (50 µl) was mixed with 50 µl of CTC prewarmed to 37°C in a clean Eppendorf tube; after 30 s, 2 µl of 12.5% paraformaldehyde in 0.5 M Tris buffer (final pH 7.4) was added and mixed well. Samples were examined using BX51 fluorescence microscopy (Olympus) with 10× ocular and 100× objective (oil immersion lens) lenses. The UV light passed through a band-pass filter of 400–440 nm with a reflector of 475 nm. In each sample, at least 300 cells were assessed for CTC staining patterns. There were three main patterns of CTC fluorescence that could be identified: F, with uniform fluorescence over the entire head, characteristic of uncapacitated cells with intact acrosomes; B, with a fluorescence-free band in the post-acrosomal region, characteristic of capacitated, acrosome-intact cells; and AR, with dull or absent fluorescence over the sperm head, characteristic of capacitated, acrosome-reacted cells. Bright fluorescence was visible on the midpiece at all three stages.

### *In vitro* Fertilization Assays

In vitro fertilization was performed as previously described with light modifications [2], [32], [46]. Mature C57 (>6 weeks of age) female mice were induced to superovulate by intraperitoneal injection of 5 IU pregnant mare serum gonadotropin (PMSG) followed by 10 IU of hCG after 48 h. The female mice were then euthanized 13 h after the hCG injection. Oviducts were collected in a 35-mm dish containing human tubal fluid (HTF) medium. The cumulus-oocyte cells were obtained by gentle dissection of the oviducts. The sperm were collected and treated by TPPII antagonists for one hour, washed twice and centrifuged at 300 g for 2 min, and added to the fertilization droplet containing the eggs. After one hour of incubation at 37°C in 5% CO_2_, the eggs were washed free of unbound sperm, transferred to droplets of the same medium and returned to the incubator. Fertilization was assessed by recording the number of two-cell embryos 24 h after insemination.

## Supporting Information

Figure S1 **Localization of TPPII protein in mouse sperm.** Cauda sperm before (0 h) and after (2 h) capacitation were probed with anti-TPPII polyclonal antibodies. Control sperm (Co) were examined by anti-TPPII polyclonal antibodies which were pre-incubated with the corresponding antigen peptide. Sperm DNA was stained with propidium iodide (PI) and can be seen in red (bars 10 µm). A representative of three independent experiments is shown.
(TIF)

Figure S2 **Localization of RyR_3_ protein in spermatozoa.** Spermatozoa were probed with (Exp) anti-RyR_3_ and (Co) anti-RyR_3_ pre-incubated with the corresponding antigen peptide. Sperm DNA was stained with propidium iodide (PI) and can be seen in red (bars 10 µm). A representative experiment of three experiments is shown.
(TIF)

Figure S3 **Proposed mechanisms by which TPPII antagonists regulated sperm function by modulating intracellular Ca^2+^ stores via ryanodine receptor 3.** The inhibition of TPPII by AAF-CMK and butabindide resulted in the activation of cAMP/PKA-mediated protein tyrosine phosphorylation. This action of TPPII antagonists was dependent on extracellular HCO_3_^–^ and BSA. Ryanodine receptor inhibitors but not IP_3_R inhibitors could block this TPPII antagonist-induced sperm protein tyrosine phosphorylation.
(TIF)

Figure S4 **Effect of EGTA on TPPII antagonist-induced changes of sperm function.** (A–C) Spermatozoa were treated with AAF-CMK (Ac, 6 µM) and butabindide (Bt, 1000 µM) for 60 min in the absence of 1 mM CaCl_2_ and in the presence of EGTA at the dose of 25 and 50 µM. The percentage (A), VAP (B), and VCL (C) of sperm motility were examined using CASA. Results are expressed as the mean ± SEM (n = 4). *P<0.05 as compared with the corresponding control (Co) (unpaired *t* test). (D) Sperm were treated with AAF-CMK (Ac, 6 µM) and butabindide (Bt, 1000 µM) for 60 min in the absence of 1 mM CaCl_2_ and in the presence of EGTA at the dose of 25 and 50 µM. Protein tyrosine phosphorylation was assessed by Western blot analysis. α-Tubulin was used as the loading control. The Western blot is a representative of four independent experiments.
(TIF)

Figure S5 **Effect of BAPAT-AM on TPPII antagonist-induced changes of sperm function.** (A–C) Spermatozoa were treated with AAF-CMK (Ac, 6 µM) and butabindide (Bt, 1000 µM) for 60 min in the absence of 1 mM CaCl_2_ and in the presence of BAPAT-AM at the dose of 2.5, 10 and 25 µM. The percentage (A), VAP (B), and VCL (C) of sperm motility were examined using CASA. Results are expressed as the mean ± SEM (n = 4). *P<0.05 as compared with the corresponding control (Co) (unpaired *t* test). (D) Sperm were treated with AAF-CMK (Ac, 6 µM) and butabindide (Bt, 1000 µM) for 60 min in the absence of 1 mM CaCl_2_ and in the presence of BAPAT-AM at the dose of 2.5, 10 and 25 µM. Protein tyrosine phosphorylation was assessed by Western blot analysis. α-Tubulin was used as the loading control. The Western blot is a representative of four independent experiments.
(TIF)
