# Supplementary figures and images for: Tripeptidyl Peptidase II Regulates Sperm Function by Modulating Intracellular Ca2+ Stores via the Ryanodine Receptor
Source: PLoS One. 2013 Jun 20;8(6):e66634. doi: 10.1371/journal.pone.0066634 (PMC3688596; doi:10.1371/journal.pone.0066634)

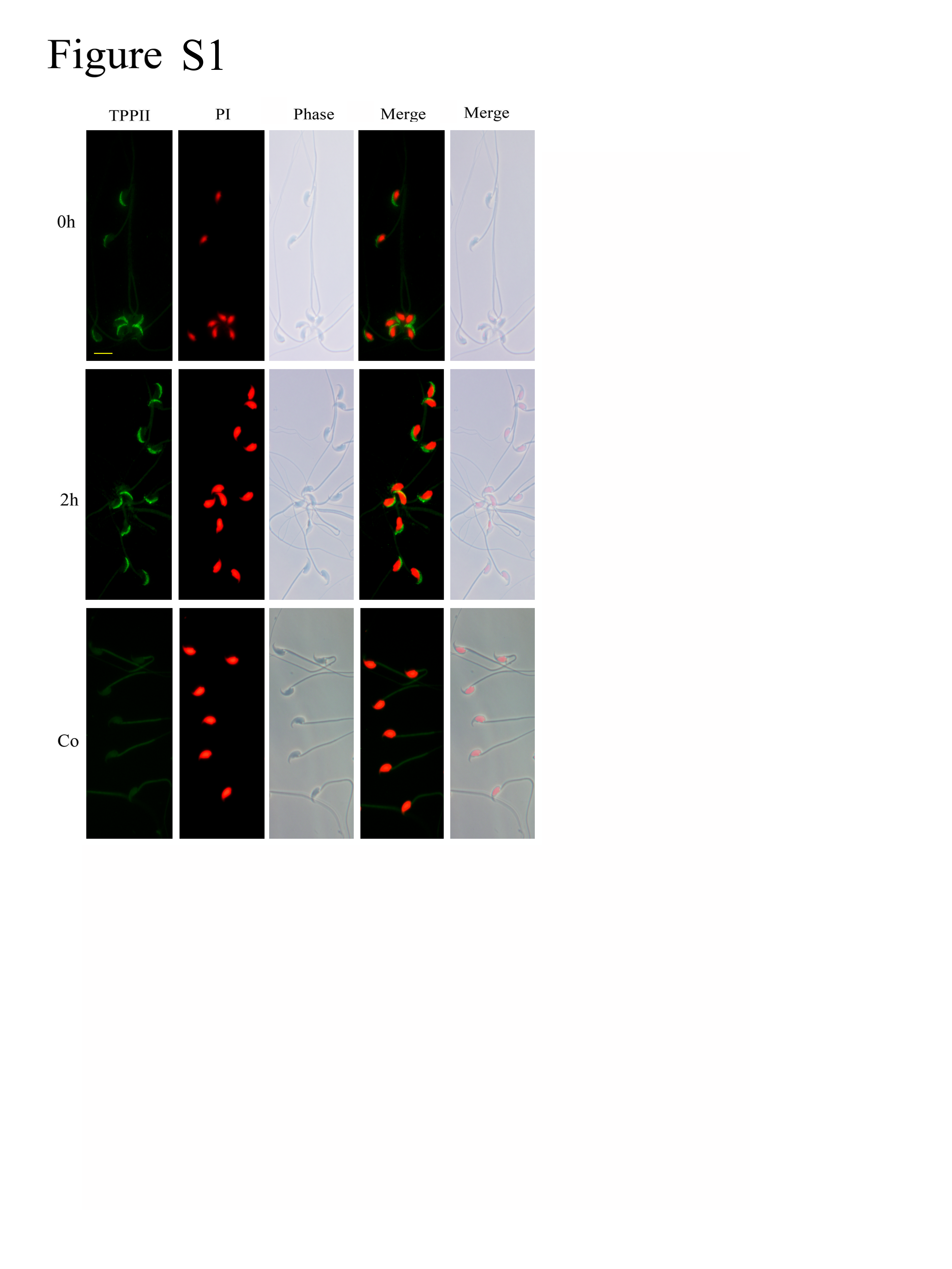

Supplement: Figure S1 — Localization of TPPII protein in mouse sperm. Cauda sperm before (0 h) and after (2 h) capacitation were probed with anti-TPPII polyclonal antibodies. Control sperm (Co) were examined by anti-TPPII polyclonal antibodies which were pre-incubated with the corresponding antigen peptide. Sperm DNA was stained with propidium iodide (PI) and can be seen in red (bars 10 µm). A representative of three independent experiments is shown. (TIF) [file pone.0066634.s001.tif]

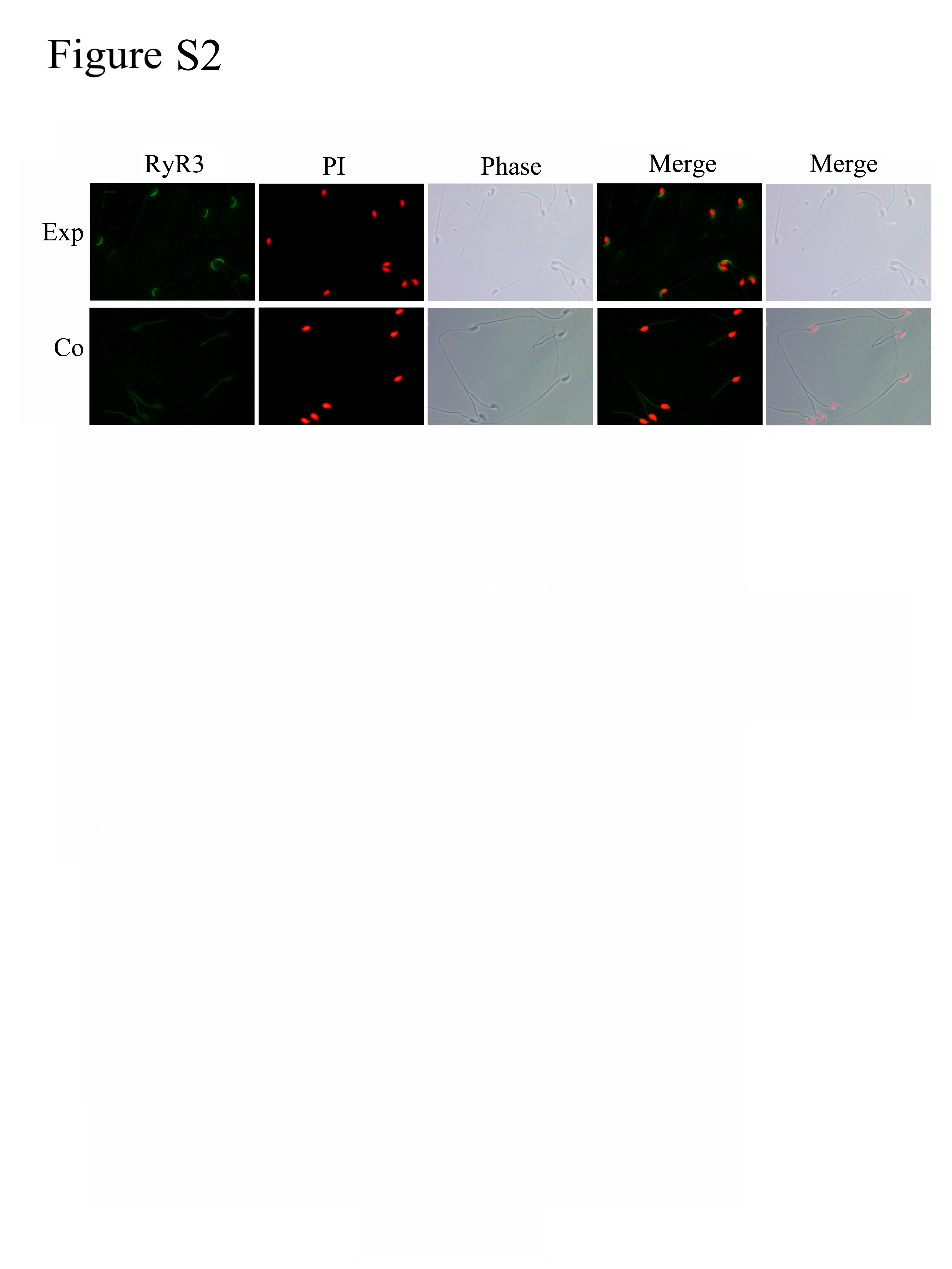

Supplement: Figure S2 — Localization of RyR3 protein in spermatozoa. Spermatozoa were probed with (Exp) anti-RyR3 and (Co) anti-RyR3 pre-incubated with the corresponding antigen peptide. Sperm DNA was stained with propidium iodide (PI) and can be seen in red (bars 10 µm). A representative experiment of three experiments is shown. (TIF) [file pone.0066634.s002.tif]

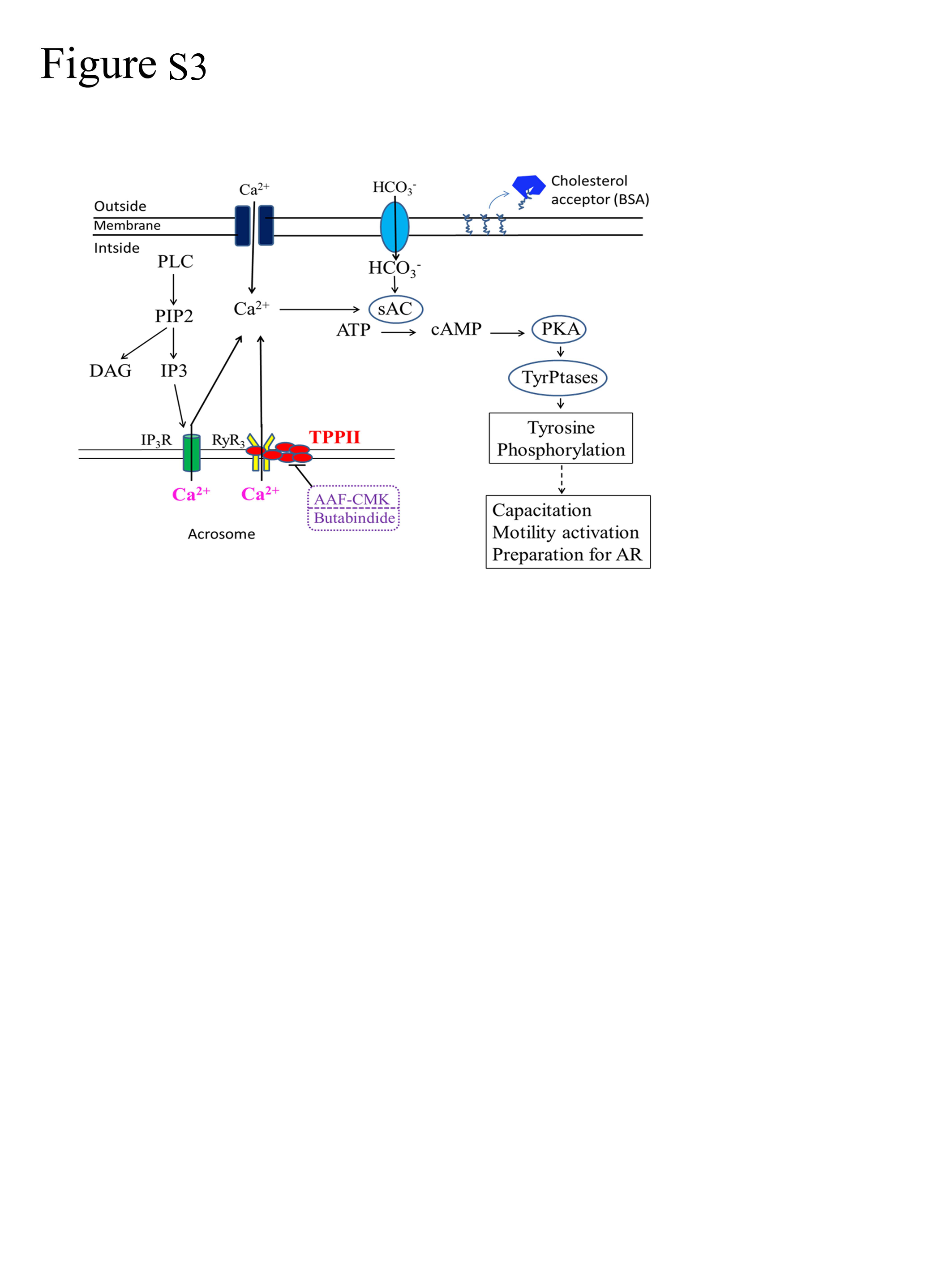

Supplement: Figure S3 — Proposed mechanisms by which TPPII antagonists regulated sperm function by modulating intracellular Ca2+ stores via ryanodine receptor 3. The inhibition of TPPII by AAF-CMK and butabindide resulted in the activation of cAMP/PKA-mediated protein tyrosine phosphorylation. This action of TPPII antagonists was dependent on extracellular HCO3 – and BSA. Ryanodine receptor inhibitors but not IP3R inhibitors could block this TPPII antagonist-induced sperm protein tyrosine phosphorylation. (TIF) [file pone.0066634.s003.tif]

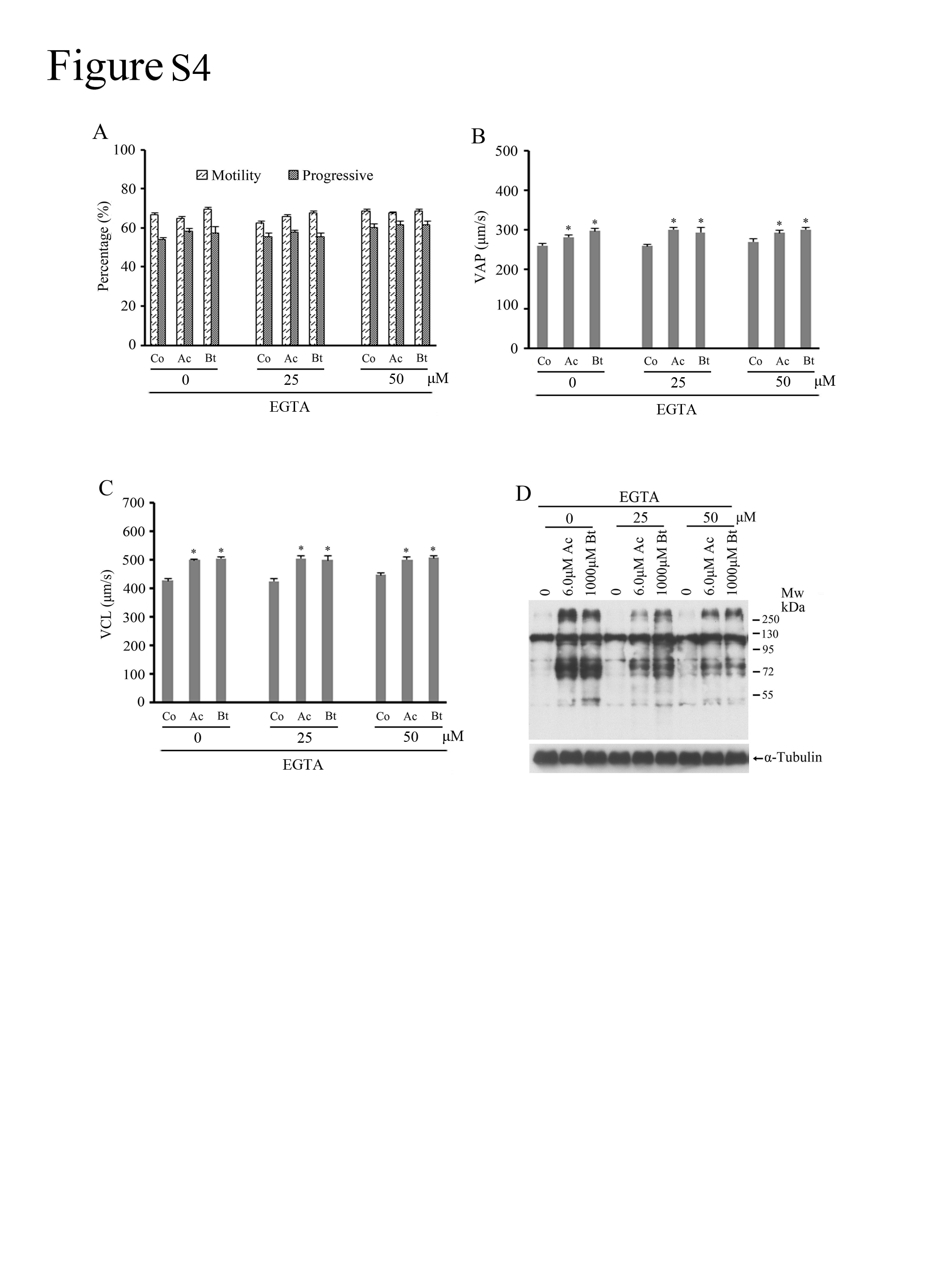

Supplement: Figure S4 — Effect of EGTA on TPPII antagonist-induced changes of sperm function. (A–C) Spermatozoa were treated with AAF-CMK (Ac, 6 µM) and butabindide (Bt, 1000 µM) for 60 min in the absence of 1 mM CaCl2 and in the presence of EGTA at the dose of 25 and 50 µM. The percentage (A), VAP (B), and VCL (C) of sperm motility were examined using CASA. Results are expressed as the mean ± SEM (n = 4). *P<0.05 as compared with the corresponding control (Co) (unpaired t test). (D) Sperm were treated with AAF-CMK (Ac, 6 µM) and butabindide (Bt, 1000 µM) for 60 min in the absence of 1 mM CaCl2 and in the presence of EGTA at the dose of 25 and 50 µM. Protein tyrosine phosphorylation was assessed by Western blot analysis. α-Tubulin was used as the loading control. The Western blot is a representative of four independent experiments. (TIF) [file pone.0066634.s004.tif]

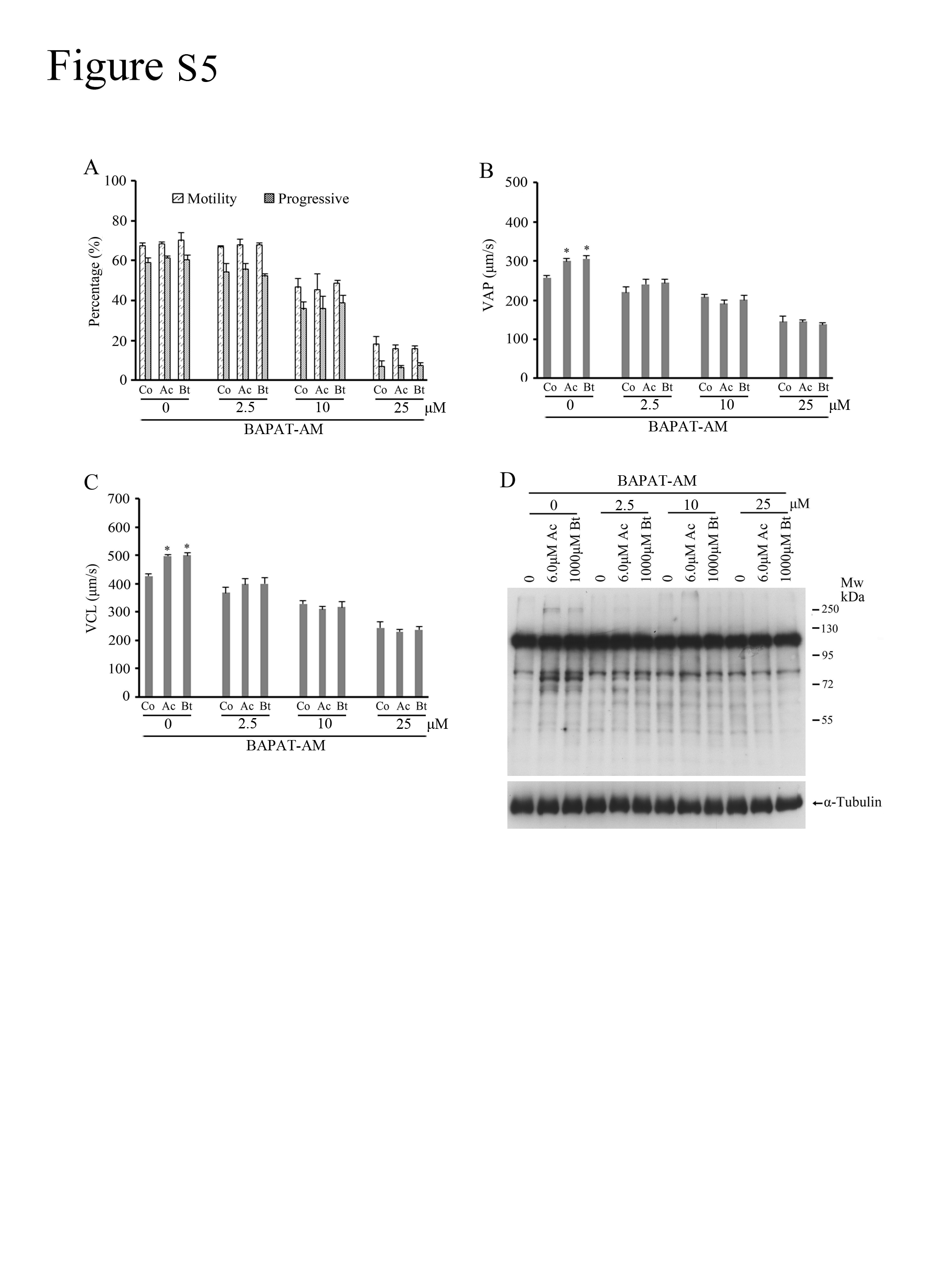

Supplement: Figure S5 — Effect of BAPAT-AM on TPPII antagonist-induced changes of sperm function. (A–C) Spermatozoa were treated with AAF-CMK (Ac, 6 µM) and butabindide (Bt, 1000 µM) for 60 min in the absence of 1 mM CaCl2 and in the presence of BAPAT-AM at the dose of 2.5, 10 and 25 µM. The percentage (A), VAP (B), and VCL (C) of sperm motility were examined using CASA. Results are expressed as the mean ± SEM (n = 4). *P<0.05 as compared with the corresponding control (Co) (unpaired t test). (D) Sperm were treated with AAF-CMK (Ac, 6 µM) and butabindide (Bt, 1000 µM) for 60 min in the absence of 1 mM CaCl2 and in the presence of BAPAT-AM at the dose of 2.5, 10 and 25 µM. Protein tyrosine phosphorylation was assessed by Western blot analysis. α-Tubulin was used as the loading control. The Western blot is a representative of four independent experiments. (TIF) [file pone.0066634.s005.tif]
